# 

**Published:** 1875-04

**Authors:** 


### CONTENTS:

ORIGINAL COMMUNICATIONS:

ART. I. Medical Expert and Medical Evidence—*concluded*. By M. A. McClelland, M.D. .... 241

ART. II. Syphilis: Its Materials and Forces. By Z. Collins McElroy, M.D., 251

ART. III. A Case of Sudden Death. By Norman Bridge, M.D. .... 276

PROGRESS IN MEDICAL SCIENCES:

Art. I. Progress of Surgery. By Jno. E. Owens, M.D. .... 279

REPORTS OF SOCIETIES:

Chicago Society of Physicians and Surgeons .... 282

CLINICS:

Lecture, by E. L. Holmes, M.D. .... 289

SELECTIONS .... 294

EDITORS' BOOK TABLE .... 300

EDITORIAL .... 310

OBITUARY .... 317

MORTALITY STATISTICS, Chicago .... 318

